# Awareness and Willingness to Use HIV Pre-Exposure Prophylaxis amongst Gay and Bisexual Men in Scotland: Implications for Biomedical HIV Prevention

**DOI:** 10.1371/journal.pone.0064038

**Published:** 2013-05-17

**Authors:** Ingrid Young, Jessica Li, Lisa McDaid

**Affiliations:** Social and Public Health Sciences Unit, Medical Research Council, Glasgow, United Kingdom; Indiana University, United States of America

## Abstract

**Objectives:**

To investigate the awareness of, and willingness to use, HIV Pre-Exposure Prophylaxis (PrEP), and willingness to take part in a PrEP study among gay and bisexual men in Scotland.

**Methods:**

Cross-sectional survey of 17 gay commercial venues in Glasgow and Edinburgh in May 2011 (N = 1515, 65.2% response rate); 1393 are included in the analyses.

**Results:**

Just under one-third of participants had heard of PrEP (n = 434; 31.2%), with awareness associated with being aged older than 35 years, talking to UAI partners about HIV, and with having had an HIV or STI test in the previous 12 months. Around half were willing to take part in a PrEP study (n = 695; 49.9%) or to take PrEP on a daily basis (n = 756; 54.3%). In multivariate analysis, willingness to take PrEP was associated with lower levels of education, regular gay scene attendance, ‘high-risk’ unprotected anal intercourse (UAI) and testing for HIV or STI in the previous 12 months. Reasons for not wanting to participate in a PrEP study or take PrEP included perceptions of low personal risk of HIV and concerns with using medication as an HIV prevention method.

**Conclusions:**

There is a willingness to engage in new forms of HIV prevention and research amongst a significant number of gay and bisexual men in Scotland. Future biomedical HIV interventions need to consider the links between sexual risk behaviour, testing, and potential PrEP use.

## Introduction

Recent research has demonstrated that prescribing antiretrovirals (ARVs) to HIV negative people before sexual exposure to HIV can help reduce HIV transmission. iPrEX demonstrated a 44% reduction in the acquisition of HIV amongst serodiscordant men who have sex with men (MSM) in couples when using Pre-exposure Prophylaxis (PrEP) [1]. The Partners Prevention study subsequently demonstrated a reduction of up to 75% amongst serodiscordant heterosexual couples [2]. The US Food and Drug Administration (FDA) approved the use of Truvada (emtricitabine/tenofovir disoproxil fumarate) for use as PrEP in July 2012, [3] and the Center for Disease Control (CDC) has issued interim guidance for both MSM and for heterosexual serodiscordant couples [4], [5]. Although there is still much debate around the efficacy and safety of PrEP, [6] the recent FDA decision to expand the indications for Truvada use in the United States has introduced a significant new biomedical option into HIV prevention. In the UK, PrEP is currently not regulated for use. Recent guidelines from the British HIV Association (BHIVA) have urged caution, given mixed findings from other PrEP trials, and further evidence that PrEP is effective and appropriate for HIV prevention in the UK is being sought [6].

MSM have been identified as a high-risk group for which PrEP may be an appropriate prevention option. There has been growth in recent research examining attitudes, awareness and willingness to use PrEP among MSM primarily in the US, [7], [8], [9], [10], [11], [12] with more limited research in Australia, China, France, Thailand and Canada [13], [14], [15], [16], [17]. To date, only one peer-reviewed study with MSM in the UK has been published, [18] although preliminary findings from a few UK studies have been reported [19], [20]. Most of these studies show very low levels of awareness of PrEP amongst MSM, but a relatively high level of willingness to use PrEP as part of an HIV prevention strategy. Although findings from these community survey studies cannot predict actual, future behaviour, they have revealed significant proportions of MSM who would consider using PrEP if available. This paper presents findings from the 2011 Medical Research Council (MRC) Gay Men’s Sexual Health Survey around attitudes towards PrEP amongst gay and bisexual men in Scotland. We examine the factors associated with awareness of, and the likelihood of taking, PrEP and consider the implications for future PrEP and HIV prevention research.

## Methods

The MRC Gay Men’s Sexual Health Survey collected anonymous, self-complete questionnaires and oral fluid specimens (collected with OraSure® Oral Specimen Collection Devices, OraSure Technologies, Inc, Bethlehem, PA) in 17 gay commercial venues (15 bars and 2 saunas) in Glasgow and Edinburgh in May 2011. A form of time and location sampling was used to recruit representative samples of MSM [21]. A team of temporary fieldworkers was trained then employed to distribute and collect questionnaires at two different time points in the early (19.00–21.00) and late evening (21.00–23.00). No bar was visited twice in the same evening. At the end of the survey each bar had been visited at both time points on each day of the week. Each sauna was visited six times over the course of the two week survey period; for a two hour period between 17∶00–19∶00 on Thursdays and between 14∶00–17∶00 on Saturdays and Sundays. A total of 2325 eligible men were approached and 1515 participated in the survey (65.2% response rate [RR]); 1218 provided oral fluid samples (52.4% RR). Among the men who declined to participate, temporary fieldworkers estimated that 28.6% were aged <30 years, 26.7% were aged 30–39 years, and 35.9% were aged 40+ years, which suggests non-participants were considerably older than men who chose to participate in the survey; 20.3% were alone in the venue at the time, 37.6% were with one other person, and 33.5% were part of a larger group. Ethical approval was granted by University of Glasgow College of Social Sciences Ethics Committee.

Questionnaires included demographics (age, area of residence, education, frequency of gay scene use), sexual health (HIV/STI testing), and sexual behaviour in the previous 12 months. HIV treatment optimism was measured via agreement with the statement ‘I am less worried about HIV infection now that treatments have improved’ (5 point Likert scale strongly agree to strongly disagree). A measure of unprotected anal intercourse (UAI), which presented a higher risk of HIV infection, was created and includes men who reported any of the three following behaviours: UAI with 2 or more partners, UAI with casual partners, and/or UAI with unknown/discordant partners in the previous 12 months.

A brief description of PrEP was provided: *Researchers are testing pre-exposure prophylaxis (PrEP) – taking antiretroviral pills daily to help prevent HIV in HIV-uninfected people at high risk. This is different from PEP which is taken AFTER exposure.* Men were asked about their awareness of PrEP (response: yes, no or don’t know), their willingness to take part in a PrEP research study (response: yes, no or don’t know), and their likelihood of taking PrEP on a daily basis if it were to become available (response: 5 point Likert scale very likely to very unlikely). Responses for awareness were recoded into two categories: had not/did not know if heard of PrEP and heard of PrEP. Responses for willingness to be a part of a research study on PrEP were recoded into uncertain/unwilling to take part in study and willing to take part in study, and responses for likelihood of using PrEP on a daily basis were recoded into unlikely/uncertain to use PrEP (very unlikely, unlikely, and uncertain combined) and likely to use PrEP (very likely and likely combined). If participants reported being uncertain or unwilling/unlikely to take part in a PrEP research study or to take PrEP on a daily basis, they were given the opportunity to answer an open ended question asking their reason for their response.

Data were analysed using SPSS 15.0 for Windows. Men who tested HIV-positive from the study using the OraSure® test and were aware of their status (n = 44) and men who had missing data on any outcome variables (n = 78) were excluded from the analyses, leaving a sample of 1393 men. This included 1108 HIV-negative men, 13 undiagnosed HIV-positive men, and 272 men who did not provide oral fluid samples (of whom, 228 reported ever HIV testing with 211 reporting the result to be HIV-negative and only 9 reported being HIV-positive). Chi-square tests were used for bivariate comparisons. Variables significant at the bivariate level (p<0.05) were entered into three logistic regression models used to estimate odds ratios (OR) and 95% confidence intervals (CI) for awareness of PrEP, willingness to participate in a PrEP research study, and likelihood of using PrEP on a daily basis.

A thematic analysis was used to examine reasons for reporting uncertainty or unwillingness to take part in a research study on PrEP or to use it on a daily basis. A total of 180 men provided short, open-ended reasons for why they were uncertain or unwilling to taking part in a study, and 195 men reported reasons for being uncertain or unlikely to take PrEP on a daily basis. All of the responses were analysed. The first author (IY) coded these short answers using an inductive approach and grouped them according to themes that emerged from the data. The second author (JL) confirmed the codings and groupings for accuracy and consistency [22], [23], [24].

## Results

### Sample Characteristics

The characteristics of the sample are shown in Table 1. The mean age of men sampled was 32.8 years (range 18–83, SD = 10.88) and nearly half had degree or postgraduate level education. The majority were frequent visitors of the gay scene. One–third reported having any higher risk UAI in the previous 12 months and 27.6% had always or sometimes talked to their yearly UAI partners about HIV. More than half had had a recent HIV or STI test and the majority were uncertain or not optimistic about HIV treatment.

**Table 1:** Sample Characteristics (N = 1393).

	n	%
**Age**		
18–25	434	31.4
26–35	454	32.9
36–45	297	21.5
46+	196	14.2
**Postcode**		
Scottish	207	15.8
Edinburgh	439	33.5
Glasgow	587	44.8
Rest of UK	78	5.9
**Qualifications**		
Secondary	192	14.7
Further/vocational	492	37.7
Degree/Postgraduate	622	47.6
**Frequency of gay scene use**		
Once a month or less	411	29.7
2–3 times a month	355	25.7
Once or more a week	616	44.6
**Had any higher-risk UAI** *		
No	918	66.6
Yes	460	33.4
**Talked about HIV with UAI partners**		
Did not have UAI in the previous 12 months	783	56.2
Always/sometimes	385	27.6
Never	225	16.2
**Had an STI in the previous 12 months**		
No	1214	88.3
Yes	161	11.7
**Had an HIV or STI test in the previous 12 months**		
No	587	43.3
Yes	768	56.7
**HIV treatment optimism** †		
Agree	278	20.1
Uncertain/disagree	1107	79.9
**Heard of PrEP**		
Yes	434	31.2
No	832	59.7
Don’t know	127	9.1
**Willingness to take part in PrEP study**		
Yes	695	49.9
No	393	28.2
Don’t Know	305	21.9
**Likelihood of using PrEP on a daily basis**		
Very likely	457	32.8
Likely	299	21.5
Uncertain	356	25.6
Unlikely	188	13.5
Very Unlikely	93	6.7

* UAI with 2 or more partners, UAI with casual partners, and/or UAI with unknown/discordant partners in the previous 12 months.

† HIV treatment optimism – ‘I am less worried about HIV infection now that treatments have improved’.

### Awareness and Willingness to use PrEP

Four hundred and thirty four men (31.2%) had heard of PrEP. Half of the men sampled were willing to take part in a research study on PrEP; a little more than one quarter did not want to and a little more than one fifth did not know if they would want to take part in a PrEP study. Similar results were reported for the likelihood of using PrEP on a daily basis to prevent HIV infection. Half of men sampled reported being likely to take it on a daily basis, while one quarter was uncertain and one fifth reported being unlikely to use it (Table 1). Willingness to take part in a PrEP research study and likelihood of using PrEP were highly associated. Among men who reported they would be unwilling to take part in a research study, 66.9% (n = 467) also reported that they would be unlikely to use PrEP on a daily basis (χ^2^ = 252.80, p<0.001).

### Factors Associated with Awareness and Willingness to use PrEP

Tables 2, 3 and 4 show the factors associated with awareness of PrEP, willingness to participate in a PrEP research study and likelihood of using PrEP on a daily basis. When controlling for the factors significant at the bivariate level in multivariate logistic regression, the odds of having heard of PrEP remained significantly higher for men aged 36+ compared to men aged 18–25, men who always or sometimes talked to their UAI partners about HIV compared to men who never did so, and men who had an HIV or STI test in the previous 12 months compared to those who did not (Table 2). The adjusted odds of being willing to participate in a research study remained significantly higher for men who visited the gay scene once or more a week compared to men who visited once a month or less, and for men who had heard of PrEP; the odds remained significantly lower for men who resided outside of Scotland than within Scottish areas and men who were uncertain or not optimistic about HIV treatment (Table 3). The adjusted odds of being likely to use PrEP on a daily basis were higher for men who had secondary or further/vocational level education compared to degree or postgraduate level education, visited the gay scene at least 2–3 times a month or more compared to once a month or less, reported any higher-risk UAI, had an HIV or STI test in the previous 12 months, and had heard of PrEP; the odds remained lower for men who were aged 26+ compared to men aged 18–25, men who resided within Edinburgh compared with elsewhere in Scotland, other than Glasgow, and men who were uncertain or not optimistic about HIV treatment (Table 4).

**Table 2:** Factors associated with Awareness of Pre-exposure Prophylaxis for HIV prevention (N = 1393).

	Did not/Did not know if heard of prep (n = 959),n (%)	Heard of PrEP(n = 434),n (%)	OR	95% CI	p value	AOR	95% CI	p value
**Age**								
18–25	320 (73.7)	114 (26.3)	1			1		
26–35	322 (70.9)	132 (29.1)	1.15	(0.86–1.55)	0.350	1.13	(0.83–1.54)	0.425
36–45	180 (60.6)	117 (39.4)	1.83	(1.33–2.50)	<0.001	1.89	(1.35–2.63)	<0.001
46+	129 (65.8)	67 (34.2)	1.46	(1.01–2.10)	0.043	1.52	(1.03–2.24)	0.034
**Postcode**								
Scottish	148 (71.5)	59 (28.5)	1					
Edinburgh	298 (67.9)	141 (32.1)	1.19	(0.83–1.71)	0.354			
Glasgow	403 (68.7)	184 (31.3)	1.15	(0.81–1.62)	0.445			
Rest of UK	50 (64.1)	28 (35.9)	1.41	(0.81–2.44)	0.228			
**Qualifications**								
Secondary	140 (72.9)	52 (27.1)	0.69	(0.48–0.99)	0.045	0.76	(0.52–1.11)	0.156
Further/vocational	350 (71.1)	142 (28.9)	0.76	(0.59–0.98)	0.033	0.81	(0.62–1.06)	0.122
Degree/Postgraduate	405 (65.1)	217 (34.9)	1			1		
**Frequency of gay scene use**								
Once a month or less	288 (70.1)	123 (29.9)	1					
2–3 times a month	251 (70.7)	104 (29.3)	0.97	(0.71–1.32)	0.849			
Once or more a week	412 (66.9)	204 (33.1)	1.16	(0.89–1.52)	0.282			
**Had any higher–risk UAI** *								
No	629 (68.5)	289 (31.5)	1					
Yes	319 (69.3)	141 (30.7)	0.96	(0.76–1.23)	0.754			
**Talked about HIV with UAI partners**								
Did not have UAI in previous 12 months	542 (69.2)	241 (30.8)	1.31	(0.94–1.84)	0.115	1.22	(0.86–1.74)	0.262
Always/sometimes	249 (64.7)	136 (35.3)	1.61	(1.12–2.32)	0.011	1.48	(1.01–2.16)	0.043
Never	168 (74.7)	57 (25.3)	1			1		
**Had an STI in the previous 12 months**								
No	848 (69.9)	366 (30.1)	1			1		
Yes	97 (60.2)	64 (39.8)	1.53	(1.09–2.15)	0.014	1.36	(0.95–1.94)	0.093
**Had an HIV or STI test in the previous 12 months**								
No	439 (74.8)	148 (25.2)	1			1		
Yes	491 (63.9)	277 (36.1)	1.67	(1.32–2.12)	<0.001	1.67	(1.29–2.15)	<0.001
**HIV treatment optimism** †								
Agree	199 (71.6)	79 (28.4)	1					
Uncertain/disagree	754 (68.1)	353 (31.9)	1.18	(0.88–1.57)	0.264			

OR = odds ratio; AOR = adjusted odds-ratio; 95% CI = 95% confidence interval.

* UAI with 2 or more partners, UAI with casual partners, and/or UAI with unknown/discordant partners in the previous 12 months.

† HIV treatment optimism 1– ‘I am less worried about HIV infection now that treatments have improved’.

**Table 3:** Factors associated with willingness to be part of a research study on Pre-exposure Prophylaxis for HIV prevention (N = 1393).

	Uncertain/unwilling to take part in a study (n = 698),n (%)	Willing to take partin a study(n = 695),n (%)	OR	95% CI	p value	AOR	95% CI	p value
**Age**								
18–25	212 (48.8)	222 (51.2)	1					
26–35	215 (47.4)	239 (52.6)	1.06	(0.82–1.38)	0.657			
36–45	165 (55.6)	132 (44.4)	0.76	(0.57–1.03)	0.075			
46+	101 (51.5)	95 (48.5)	0.90	(0.64–1.26)	0.533			
**Postcode**								
Scottish	88 (42.5)	119 (57.5)	1			1		
Edinburgh	225 (51.3)	214 (48.7)	0.70	(0.50–0.98)	0.038	0.72	(0.51–1.03)	0.070
Glasgow	284 (48.4)	303 (51.6)	0.80	(0.57–1.09)	0.146	0.81	(0.58–1.13)	0.205
Rest of UK	48 (61.5)	30 (38.5)	0.46	(0.27–0.79)	0.005	0.54	(0.31–0.94)	0.029
**Qualifications**								
Secondary	86 (44.8)	106 (55.2)	1.48	(1.07–2.04)	0.019	1.31	(0.92–1.86)	0.133
Further/vocational	233 (47.4)	259 (52.6)	1.33	(1.05–1.69)	0.018	1.18	(0.92–1.52)	0.186
Degree/Postgraduate	339 (54.5)	283 (45.5)	1			1		
**Frequency of gay scene use**								
Once a month or less	235 (57.2)	176 (42.8)	1			1		
2–3 times a month	180 (50.7)	175 (49.3)	1.30	(0.98–1.73)	0.073	1.26	(0.94–1.70)	0.123
Once or more a week	276 (44.8)	340 (55.2)	1.65	(1.28–2.12)	<0.001	1.55	(1.19–2.02)	0.001
**Had any higher–risk UAI** *								
No	506 (55.1)	412 (44.9)	1			1		
Yes	187 (40.7)	273 (59.3)	1.79	(1.43–2.25)	<0.001	1.39	(0.96–2.03)	0.085
**Talked about HIV with UAI partners**								
Did not have UAI in previous 12 months	429 (54.8)	354 (45.2)	0.58	(0.43–0.79)	0.001	0.82	(0.53–1.28)	0.382
Always/sometimes	176 (45.7)	209 (54.3)	0.84	(0.60–1.17)	0.313	0.91	(0.64–1.30)	0.596
Never	93 (41.3)	132 (58.7)	1			1		
**Had an STI in the previous 12 months**								
No	621 (51.2)	593 (48.8)	1			1		
Yes	68 (42.2)	93 (57.8)	1.43	(1.03–2.00)	0.034	1.08	(0.75–1.54)	0.684
**Had an HIV or STI test in the previous 12 months**								
No	318 (54.2)	269 (45.8)	1			1		
Yes	359 (46.7)	409 (53.3)	1.35	(1.09–1.67)	0.007	1.24	(0.98–1.56)	0.074
**HIV treatment optimism** †								
Agree	111 (39.9)	167 (60.1)	1			1		
Uncertain/disagree	585 (52.8)	522 (47.2)	0.59	(0.45–0.78)	<0.001	0.62	(0.47–0.82)	<0.001
**Heard of PrEP**								
No/Don’t know	504 (52.6)	455 (47.4)	1			1		
Yes	194 (44.7)	240 (55.3)	1.37	(1.09–1.72)	0.007	1.39	(1.09–1.77)	0.008

OR = odds ratio; AOR = adjusted odds-ratio; 95% CI = 95% confidence interval.

* UAI with 2 or more partners, UAI with casual partners, and/or UAI with unknown/discordant partners in the previous 12 months.

† HIV treatment optimism – ‘I am less worried about HIV infection now that treatments have improved’.

**Table 4:** Factors associated with likelihood of using Pre-exposure Prophylaxis for HIV prevention (N = 1393).

	Unlikely/uncertainto use PrEP (n = 637),n (%)	Likely to use PrEP(n = 756),n (%)	OR	95% CI	p value	AOR	95% CI	p value
**Age**								
18–25	156 (35.9)	278 (64.1)	1			1		
26–35	223 (49.1)	231 (50.9)	0.58	(0.44–0.76)	<0.001	0.72	(0.54–0.96)	0.025
36–45	152 (51.2)	145 (48.8)	0.54	(0.40–0.72)	<0.001	0.67	(0.48–0.93)	0.016
46+	101 (51.5)	95 (48.5)	0.53	(0.38–0.74)	<0.001	0.60	(0.42–0.88)	0.008
**Postcode**								
Scottish	78 (37.7)	129 (62.3)	1			1		
Edinburgh	214 (48.7)	225 (51.3)	0.64	(0.45–0.89)	0.009	0.68	(0.48–0.98)	0.041
Glasgow	262 (44.6)	325 (55.4)	0.75	(0.54–1.04)	0.083	0.79	(0.56–1.11)	0.177
Rest of UK	43 (55.1)	35 (44.9)	0.49	(0.29–0.83)	0.008	0.65	(0.37–1.13)	0.126
**Qualifications**								
Secondary	79 (41.1)	113 (58.9)	1.63	(1.17–2.26)	0.004	1.53	(1.07–2.20)	0.020
Further/vocational	194 (39.4)	298 (60.6)	1.75	(1.38–2.22)	<0.001	1.49	(1.15–1.93)	0.003
Degree/Postgraduate	331 (53.2)	291 (46.8)	1			1		
**Frequency of gay scene use**								
Once a month or less	217 (52.8)	194 (47.2)	1			1		
2–3 times a month	158 (44.5)	197 (55.5)	1.40	(1.05–1.86)	0.022	1.42	(1.05–1.92)	0.024
Once or more a week	258 (41.9)	358 (58.1)	1.55	(1.21–2.00)	0.001	1.35	(1.03–1.77)	0.029
**Had any higher–risk UAI** *								
No	482 (52.5)	436 (47.5)	1			1		
Yes	152 (33.0)	308 (67.0)	2.24	(1.77–2.83)	<0.001	2.14	(1.46–3.15)	<0.001
**Talked about HIV with UAI partners**								
Did not have UAI in previous 12 months	398 (50.8)	385 (49.2)	0.50	(0.37–0.69)	<0.001	1.00	(0.64–1.57)	0.992
Always/sometimes	162 (42.1)	223 (57.9)	0.72	(0.51–1.01)	0.056	0.78	(0.54–1.14)	0.197
Never	77 (34.2)	148 (65.8)	1					
**Had an STI in the previous 12 months**								
No	573 (47.2)	641 (52.8)	1			1		
Yes	58 (36.0)	103 (64.0)	1.59	(1.13–2.23)	0.008	1.12	(0.77–1.63)	0.547
**Had an HIV or STI test in the previous 12 months**								
No	304 (51.8)	283 (48.2)	1			1		
Yes	317 (41.3)	451 (58.7)	1.53	(1.23–1.90)	<0.001	1.38	(1.09–1.76)	0.008
**HIV treatment optimism** †								
Agree	96 (34.5)	182 (65.5)	1			1		
Uncertain/disagree	540 (48.8)	567 (51.2)	0.55	(0.42–0.73)	<0.001	0.57	(0.43–0.77)	<0.001
**Heard of PrEP**								
No/Don’t know	458 (47.8)	501 (52.2)	1			1		
Yes	179 (41.2)	255 (58.8)	1.30	(1.04–1.64)	0.024	1.38	(1.07–1.76)	0.012

OR = odds ratio; AOR = adjusted odds-ratio; 95% CI = 95% confidence interval.

* UAI with 2 or more partners, UAI with casual partners, and/or UAI with unknown/discordant partners in the previous 12 months.

† HIV treatment optimism – ‘I am less worried about HIV infection now that treatments have improved’.

### Reasons given for not Participating in a PrEP Study

Out of the 698 men who reported being unlikely or uncertain about participating in a PrEP study, 180 men (25.8%) supplied a reason in response to the open-ended question. One-quarter (n = 46) described themselves as being at low risk of HIV because of: being in a long-term, stable and/or monogamous sexual relationship; practicing safer sex; or having limited numbers of sexual partners. Of these men, 89.1% (n = 41) reported no higher-risk UAI in the previous 12 months. One-quarter (n = 45) cited concerns with using medication for HIV prevention and a further one-quarter (n = 41) reported not being interested in participating in a trial. Other reasons included: there not being enough information available on PrEP (n = 12); anxieties around privacy and/or embarrassment (n = 9); or concerns that PrEP might encourage irresponsible behaviour (n = 4).

### Reasons given for being Unlikely to use PrEP

Of the 637 men who reported being unlikely or uncertain about using PrEP, 195 men (30.6%) supplied a reason for this. Over half (n = 114) reported that they did not perceive themselves to be at risk of HIV transmission because they practiced safer sex, had limited and/or regular sexual partners or avoided high risk sexual acts. Of the men who answered in this way, 85.0% reported no higher-risk UAI in the previous 12 months. Around one-quarter of respondents (n = 51) described concerns around the medication itself. A minority (n = 7) described concerns that PrEP may encourage irresponsible sexual behaviour. There were no significant differences in age, other demographics, sexual risk behaviours or sexual health between men who reported perceptions of risk and men who reported concerns with using mediations as their reasons for being unlikely to use PrEP (data not shown).

## Discussion

This is the first study to report on awareness and willingness to use PrEP among MSM in Scotland. We found that 31.2% of participants had heard of PrEP, 49.9% were willing to take part in a PrEP study and 54.3% of participants reported being likely or very likely to take PrEP on a daily basis. These findings suggest that there is a willingness to engage in new forms of HIV prevention amongst a significant number of MSM in Scotland.

The levels of awareness in our sample appear to be much higher than those reported in other studies with MSM. A Canadian study with 195 MSM conducted in 2010 found limited knowledge (14.1%) of PrEP [17]. A more recent online survey of MSM in the US conducted after the release of the iPrEX findings in 2011 found similarly low levels of awareness (19%), [10] and an online survey of 1500 MSM in England in June 2011 found that 17% of respondents were aware of PrEP [20]. Findings from this study suggest that an increasing minority of MSM may already be aware of PrEP, which will have policy and prevention implications. There have been some concerns about possible use of PrEP off-label, and the potential problems with this mismatch of knowledge and availability of PrEP. However, a recent study found limited off-label use of PrEP (2.2%) amongst MSM in London [18]. In light of the 2012 FDA decision to approve the use of Truvada for PrEP in the United States, and subsequent international media coverage of PrEP debates, levels of PrEP awareness are likely to increase.

Levels of willingness to use PrEP in our study were similar to reported levels in both UK studies (52% & 52.4%) [18], [20]. In contrast, studies in the US found that 67% [25] of participants in California and 74% [12] of participants in Boston were willing to use PrEP while an Australian study reported only 28% willingness [13]. This variation in levels of willingness with non-UK based studies indicates the need to consider what factors contribute to potential PrEP acceptability. Furthermore, the significant result for the ‘rest of the UK’ category in the postcode variable suggests that there could be regional variations in attitudes towards biomedical prevention, as well as participating in such research.

Age appeared to be an important factor, as older men (36+ years) were more likely to have heard of PrEP. Although it is not known how participants are accessing this information, the variation in PrEP awareness between older and younger men suggests that there may be a generational difference in how men share and receive knowledge and access sexual health information [26]. Age was also associated with likelihood to use PrEP, with men aged 16–25 years being most likely to be willing to use it. While Sigma *et al* [20] found no association with age and willingness to use PrEP, Aghaizu *et al* [18] found that being aged under 35 was associated with acceptability of PrEP. The difference in associations between age groups raises a number of questions relating to how access to sexual health information, sexual behaviour and risk management may be affected by age. These findings suggest the need for age-targeted education programmes and support if PrEP is to be offered in the UK.

Men reporting HIV-related sexual risk behaviour were more than twice as likely to report willingness to use PrEP than men who did not and those tested for an STI and/or HIV in the last 12 months were almost 1.4 times as likely than men who had not tested. This may indicate not only a concern with risk of STI and/or HIV infection but also a response or management of this particular risk. Holt *et al* [13] suggest that for men in their sample who were interested in using PrEP but engaging in high-risk UAI, PrEP could represent additional HIV prevention: as these men were unlikely to use condoms, PrEP would at least act to reduce the risks of HIV transmission. Although Golub *et al* [9] point to mathematical models which demonstrate how an increase in risk behaviour could offset the benefits of PrEP, Holt’s example highlights the complexities of how PrEP might work in combination with testing for those men who are engaging in UAI. While Holt *et al*’s observation is in relation to risk compensation, an area which was not explored in our study, our findings relating to sexual behaviour and testing practices suggest the need to think about how men perceive and respond to risk in different ways and that responses to risk of HIV may not always or only involve condoms. For instance, MSM in London who had experience of using post-exposure prophylaxis (PEP) were found to be more willing to use PrEP [18]. In relation to future PrEP roll-out, these findings also suggest the need for PrEP education programmes that are linked to HIV and STI testing, which would build on existing knowledge and testing practices.

It is important to consider why nearly half of the men in our survey rejected the option of PrEP. A significant proportion reported low perceptions of risk relating to condom use, partner selection or avoiding risky sexual behaviours. This suggests a preference amongst some for behavioural HIV risk management strategies, as well as the importance of if and how risk is perceived. As has been highlighted in PrEP trials, which failed to show effectiveness, [27] perceptions of HIV risk play an important role in adherence and ultimately to the effectiveness of PrEP as a prevention method. Moreover, concerns around the use of medication for HIV prevention suggests the need to consider broader attitudes towards medication, health and HIV prevention such as poor experiences or awareness of ART in HIV positive peers and a lack of trust in the efficacy of the prevention technology of PrEP.

This study has a number of limitations to consider. The nature of this study means that it cannot predict actual, future behaviour in relation to PrEP use. However, the findings do indicate that MSM would be willing to consider using PrEP if it became available in Scotland. Caution should be taken in generalizing the findings beyond the survey population or to the wider population of non-scene going MSM. The characteristics, risk behaviours, and awareness and willingness to use PrEP among non-participants could be different from those who participated in our survey. Men who chose not to participate appeared to be older than the men who did participate and this, along with the fact that only men who attended the venues surveyed have the opportunity to participate, should be taken into consideration in the interpretation of our results. Findings also relied on self-report data which could be subject to self-report bias. However, it is hoped that the anonymous, self-complete nature of the survey limited the potential of this. In addition, the men were asked about their willingness to use a technology they knew little about. While the survey provided a brief description of what PrEP was, the description did not provide information on potential side effects, costs, efficacy or the need to maintain condom use, which could influence willingness responses. It is also possible that participants misunderstood PrEP with PEP, which is currently available and for which a campaign in Scotland had been undertaken the previous year [28]. However, the questionnaire explicitly stated that PrEP was ‘different to PEP which is taken AFTER exposure,’ in an attempt to avoid this confusion.

This study has demonstrated a strong knowledge base and interest in PrEP amongst MSM in Scotland. Findings indicate the need to build on this interest and to consider the potentially complex relationship between sexual risk behaviour, testing and interest in PrEP. Age, HIV/STI testing and perceptions of risk are factors that have been found to have a significant impact on PrEP acceptability. It will be essential for these factors to be addressed when considering for whom PrEP is best suited, and how access to and use of PrEP will be best supported. Finally, it will also be imperative to consider how risk might be managed with PrEP in combination with other behavioural HIV prevention strategies if and when PrEP becomes available in the UK.
